# Commentaries on Viewpoint: Do we need to change the guideline values for determining low bone mineral density in athletes?

**DOI:** 10.1152/japplphysiol.00203.2022

**Published:** 2022-05-01

to the editor: The Viewpoint by Jonvik et al. (1) proposes to change the guidelines to interpret athletes’ bone mineral density (BMD) *Z*-scores in low- versus high-impact sports. This proposal is mainly based on the potential role of BMD as a marker of low energy availability (LEA)/relative energy deficiency in sport (RED-S). It can be argued, however, that the primary function of BMD measurements is to analyze the risk of (osteoporotic) fractures in an individual (2). In this regard, BMD *Z*-scores seem to be related to (stress) fracture risk, independent of sports discipline (3). Furthermore, achieving a high peak bone mass, and preventing low absolute BMD, in early adulthood may prevent osteoporosis and fractures later in life (4,5). These arguments do not support the use of sport-specific guidelines for the interpretation of BMD *Z*-scores. Nevertheless, we agree with Jonvik et al. (1) that high-impact sports could mask the presence of LEA/RED-S when using population-based *Z*-scores. Some potential pitfalls of this approach should be noted as well. First, the distinction between low- and high-impact sports is not always clear because low-impact sports can also include high-impact training. Second, sport-specific impact can differentially affect BMD status at various measurement sites. Finally, BMD changes occur slowly, which complicates the early detection and treatment of LEA by this instrument. In conclusion, sport-specific reference values could be a valuable adjunct in the diagnosis of LEA, but probably less important with regard to (stress) fracture risk. For early detection and prevention of LEA, research might need to focus more on the validation and use of objective blood markers.

## DISCLOSURES

No conflicts of interest, financial or otherwise, are declared by the authors.
