# Supplementary material for: Commentaries on Viewpoint: Do we need to change the guideline values for determining low bone mineral density in athletes?
Source: J Appl Physiol (1985). 2022 May 1;132(5):1323–4. doi: 10.1152/japplphysiol.00203.2022 (PMC9208432; doi:10.1152/japplphysiol.00203.2022)
Supplement: Related texts

# Article commentary: *Z*-scores for athletes: The need of transition from blanket to bracket approach

**Authors:** Shival Srivastav, Mahesh Arjundan Gadhvi

**Affiliations:** Department of Physiology, All India Institute of Medical Sciences, Jodhpur, India

to the editor: The effect of physical activity on bone mineral density (BMD) is not uniform across different sports (1). The net “osteogenic” effect of different sports is multifactorial and may be contingent on diverse physiological factors including, but not limited to, age and gender (2). In addition, genetic factors may also be at play. Protracted stretches of low energy availability (LEA) following engagement in sports may compound the problem (3,4), predisposing athletes to risk of osteoporosis and stress fractures.

In light of the present information, a blanket approach to identify athletes at risk of reduced bone mineral density (BMD) needs to be repudiated. There is a strong need to define activity-specific *Z*-scores for athletes. Also, the aforementioned miscellaneous determinants should also be cogitated upon.

This will facilitate early identification of at-risk individuals and enable institution of corrective measures. As proposed by Jonvik et al. (4), this is possible only by global congregation of sports and exercise physiology experts. Their collective efforts should culminate in generation of sport/event-specific cumulative data while also factoring in the other determinants. In a nutshell, there is a need for transition from a blanket to bracket approach for risk stratification using *Z*-scores.

## DISCLOSURES

No conflicts of interest, financial or otherwise, are declared by the authors.

## References

[1] Scofield KL, Hecht S. Bone health in endurance athletes: runners, cyclists, and swimmers. Curr Sports Med Rep 11: 328–334, 2012. doi:10.1249/JSR.0b013e3182779193.23147022

[2] Stojanović E, Radovanović D, Dalbo VJ, Jakovljević V, Ponorac N, Agostinete RR, Svoboda Z, Scanlan AT. Basketball players possess a higher bone mineral density than matched non-athletes, swimming, soccer, and volleyball athletes: a systematic review and meta-analysis. Arch Osteoporos 15: 123, 2020. doi:10.1007/s11657-020-00803-7.32761271

[3] Logue DM, Madigan SM, Melin A, Delahunt E, Heinen M, Donnell S-JM, Corish CA. Low energy availability in athletes 2020: an updated narrative review of prevalence, risk, within-day energy balance, knowledge, and impact on sports performance. Nutrients 12: 835, 2020. doi:10.3390/nu12030835.32245088PMC7146210

[4] Jonvik KL, Torstveit MK, Sundgot-Borgen JK, Mathisen TF. Do we need to change the guideline values for determining low bone mineral density in athletes? J Appl Physiol (1985). doi:10.1152/japplphysiol.00851.2021.35060767PMC9126212

# Article commentary: Commentary on Viewpoint: Do we need to change the guideline values for determining low bone mineral density in athletes?

**Authors:** Ricardo J. Fernandes, Dalton Pessoa Filho, Maria Paula Santos

**Affiliations:** ^1^Centre of Research, Education, Innovation and Intervention in Sport, Faculty of Sport, University of Porto, Porto, Portugal; ^2^Porto Biomechanics Laboratory, Faculty of Sport, University of Porto, Porto, Portugal; ^3^Department of Physical Education, São Paulo State University, São Paulo, Brazil; ^4^Research Center in Physical Activity, Health and Leisure Faculty of Sports, University of Porto, Porto, Portugal; ^5^Laboratory for Integrative and Translational Research in Population Health, Porto, Portugal

to the editor: Jonvik et al. (1) discussed the importance of well-classifying bone mineral density (BMD) in people engaged in sports since scoring them with reference to a normal population does not seem valid. This is not the first time that the *Journal of Applied Physiology* presents commentaries on a viewpoint on BMD-related topics (2), highlighting the relevance of bone health in sports. However, we need to consider that the exposure to exercise varies according to the practitioners’ level (recreational, age-group, competitive, or elite), with the metabolic and mechanical loading affecting bone health differently due to the diverse *1*) workout volume, intensity, density, and complexity and *2*) recovery period strategies. Specifically in swimming, the voluminous general training period, the systematic exposure to the no-impact aquatic environment (training frequency is frequently greater than nine 2- to 3-h sessions/wk), and the very-high-intensity training sets of the specific and precompetition periods (3) might lead to significant low energy availability, causing low BMD. However, it is simplistic to define swimming as a low-impact sport since, in every training unit from young age, swimmers are involved in dry-land workouts aiming for strength development (4) and in in-water resistance exercises (with parachutes, drag suits, paddles, or fins). In addition, the starting and turning phases occupy ∼30% of the swimming distance total duration (5), meaning that swimmers push off the starting block and the turning wall systematically. Therefore, when developing a sport-specific BMD classification scale, sport level/expertise should be considered, and swimming (except for the open-water specialty) distinguished from other low-impact sports (e.g., cycling) and nonpractitioners.

## DISCLOSURES

No conflicts of interest, financial or otherwise, are declared by the authors.

## References

[1] Jonvik KL, Torstveit MK, Sundgot-Borgen JK, Mathisen TF. Do we need to change the guideline values for determining low bone mineral density in athletes? J Appl Physiol (1985). doi:10.1152/japplphysiol.00851.2021.35060767PMC9126212

[2] Brocherie F, Camacho-Cardenosa M, Timon R, Debevec T, Rittweger J, Hutson MJ, O’Donnell E, Brooke-Wavell K, Blagrove RC, Beck ON, Kipp S, Fernandes RJ, Conceição F, Abraldes JA, Greeves JP, O’Leary TJ, Vagula M, Gibbs JC, Churchward-Venne TA, Dolan E, Pinto RS, Oliveira LF, Sale C, Saunders B. Commentaries on Viewpoint: Fragile bones of elite cyclists: to treat or not to treat? J Appl Physiol 1985 131: 29–33, 2021. doi:10.1152/japplphysiol.00335.2021. 34181462

[3] Mujika I, Chatard J-C, Busso T, Geyssant A, Barale F, Lacoste L. Effects of training on performance in competitive swimming. Can J Appl Physiol 20: 395–406, 1995. doi:10.1139/h95-031.8563672

[4] Vorontsov A. Strength and power training in swimming. In: World Book of Swimming: From Science to Performance, edited by Seifert L, Chollet D, Mujika I. Hauppauge, New York: Nova Science Publishers, 2011, p. 313–343.

[5] Morais JE, Marinho DA, Arellano R, Barbosa TM. Start and turn performances of elite sprinters at the 2016 European Championships in swimming. Sports Biomech 18: 100–114, 2019. doi:10.1080/14763141.2018.1435713.29578384
